# Family Childcare Types and Conduct Problem Behaviors in Young Children: The Mediation Role of Caregiver-Child Interaction

**DOI:** 10.3389/fped.2018.00217

**Published:** 2018-08-06

**Authors:** Li Liu, Lijun Fan, Xiang-Yu Hou, Chuan-An Wu, Xiao-Na Yin, Guo-Min Wen, Dengli Sun, Dan-Xia Xian, Hui Jiang, Jin Jing, Yu Jin, Wei-Qing Chen

**Affiliations:** ^1^Guangzhou Key Laboratory of Environmental Pollution and Health Assessment, Guangdong Provincial Key Laboratory of Food, Nutrition and Health, Department of Medical Statistics and Epidemiology, School of Public Health, Sun Yat-sen University, Guangzhou, China; ^2^School of Public Health and Social Work, Australia China Centre for Public Health, Queensland University of Technology, Brisbane, QLD, Australia; ^3^Women's and Children's Hospital of Longhua New District of Shenzhen, Shenzhen, China; ^4^Department of Maternal and Child Health, School of Public Health, Sun Yat-sen University, Guangzhou, China; ^5^Department of Information Management, Xinhua College, Sun Yat-sen University, Guangzhou, China

**Keywords:** family childcare types, caregiver-child interaction, conduct problem behaviors, mediation, young children

## Abstract

**Background:** Previous studies have demonstrated the impacts of genetic, family, and community factors on child conduct problems (CPs). However, little is understood regarding the association between family childcare types and child conduct problem behaviors, as well as whether and to what extent caregiver-child interaction mediates the above association.

**Methods:** 9,289 children first entering kindergartens in the Longhua New District of Shenzhen, China were enrolled in this cross-sectional study. Primary caregivers were invited to fulfill a self-administered structured questionnaire containing data regarding socio-demographics, family childcare types, caregiver-child interaction, and child conduct problem behaviors (measured by the Conners' Conduct Problem Subscale). A series of multiple logistic and linear regression models were employed to assess the associations among family childcare, caregiver-child interaction, and child conduct problem behaviors.

**Results:** Family childcare types other than by parents together (i.e., mother alone, mother with others, grandparents, or changing caregivers) were all significantly associated with higher risks of conduct problem behaviors in young children (adjusted ORs ranged from 2.18 to 2.55, and adjusted βs ranged from 0.043 to 0.073; all *p* < 0.05), after adjusting for confounders including child age, gender, parental education level, parental age at pregnancy, marital status, and family income. The following family childcare types (mother alone, or grandparents, or changing caregivers) vs. the childcare by parents together showed significant relative indirect effects on conduct problem behaviors through caregiver-child interaction, indicating the significant mediation effect of caregiver-child interaction on the above associations. Mediation of caregiver-child interaction on the effect of being cared by mother with others relative to care by parents together on child conduct problem behaviors was yet non-significant.

**Conclusions:** Family childcare types other than by parents together are associated with increased risks for conduct problem behaviors in young children. Caregiver-child interaction may function as a potential mediator for the above association.

## Introduction

Conduct problems (CPs) are among the most common psychological conditions in early childhood, characterized by common externalizing behaviors including troublesome, aggression, disruption, oppositionality, delinquency, and antisociality (1–3). Children with CP diagnoses or elevated conduct problem behaviors tend to encounter increased risks for a variety of academic (e.g., school failure and dropout), social (e.g., anti-society behaviors and violent crimes), and health (e.g., substance misuse and depression) problems, and they exert enormous economic burden to the education, health, community service and justice systems (1–5). Conduct problems can occur at an age of as early as three, and early starters (onset during childhood) often exhibit more persistent and detrimental outcomes extending to adolescence and adulthood, as compared with late starters (onset from mid- to late-adolescence) (6–9). Conduct problems may lead to a lifetime of dysfunction if without effective treatment in place (1, 4, 6), which thus emphasizes the pressing need of exploring the risk factors for conduct problem behaviors in young children as well as the potential mechanisms for developing effective prevention and intervention programs.

Causes for the development and persistence of child conduct problems include a wide range of genetic and environmental factors involving child, family, and community (3, 4, 6, 10, 11), among which the early experience with family and caregivers (e.g., use of childcare) has been documented to critically affect child development (6, 11, 12). Childcare is often categorized into parental, informal (untrained grandparents, extended family, relatives, friends or employed help in the home) or formal (childcare center or nursery) care (13, 14). Previous research into the association of different childcare with children's psychological well-being has been extensive but reached controversary conclusions. For instance, several studies demonstrated a protective role of increasing quantity of parental care on cognitive outcomes or externalizing behavior problems in children (15–17). Another study, however, found that there was no increase in relative risk of severe externalizing behaviors for non-relative and center care children as compared with parental care children (14). Besides, father functioning in childcare was supported by prior research as associated with improvement for psychological, cognitive and academic development of preschool-aged children (14, 18, 19). Children receiving informal care from grandparents (20, 21) or relatives (14) were found to experience significantly more behavioral problems than their similar schoolmates. Many studies consistently showed that children who experienced multiple and concurrent childcare arrangements displayed more internalizing and externalizing behavior problems and fewer prosocial behaviors (12, 22–24). There were also a large pool of research focusing on the role of formal care, which however, also concluded inconsistent findings regarding its association with psychobehavioral outcomes in children, with some studies favoring that childcare arrangement while others not (14, 22, 25, 26). Besides, previous research largely focused on non-parental childcare (especially formal care) arrangements in Western countries, while few consideration has been given to family or informal childcare common in the rest of globe, including in Asian countries like China (27). Given the aforementioned discrepancies in the impact of childcare on psychological health as well as the scarce evidence in Chinese population which has very unique social culture, more research is thus warranted toward childcare and child development in China.

The precise mechanism of the association between childcare and child psychological development including conduct problems remains unclear. However, previous evidence has shown that the varieties of childcare arrangement are associated with different interactions between childcare and child (18, 28, 29). For example, mothers engaged in childcare often provided nurturing care and emotional support, while fathers usually spent more time in playful and physically stimulating interactions with their children (18, 30, 31). Grandparents, by contrast, were likely to focus more on children's physiological demands (e.g., dieting, sleeping) rather than active interactions (e.g., emotional support, reading, traveling) (21, 29). Different types of caregivers seemed to engage in different amount and pattern of interacting activities with children (18, 28, 29). Moreover, a growing body of research proposed that incremental involvement in caregiver-child interaction (e.g., outdoor play, bookreading, etc.) would be linked to improved cognitive and behavioral development, language acquisition, and social abilities in children (28, 32–34). It is thus of interest to test whether caregiver-child interaction may play a mediating role in the association between childcare and child development.

Referring to the aforementioned evidence, this study therefore, taking the advantage of a large population-representative sample, aims to explore the association of different family childcare types with conduct problem behaviors in young preschool children aged around three years, as well as whether and to what extent caregiver-child interaction mediates the above association. We hypothesize that: (1) different types of family childcare in China are associated with varied levels of risks for young children's behavioral problems, and childcare by parents together could be superior over other childcare types; (2) different types of family childcare have different levels of caregiver-child interaction, and an increasing caregiver-child interaction level is related to a decreased risk for child conduct problem behaviors; and (3) caregiver-child interaction serves as a mediator in the association between childcare and child behavioral development.

## Materials and methods

### Study population

Participants were recruited from the baseline survey of the Longhua Child Cohort Study (LCCS) taken place in September 2014. The LCCS was set up in the Longhua New District of Shenzhen, China, which aimed to investigate the influences of family and school environment surrounding children's early life on child psycho-behavioral development. We enrolled children aged around 3 years who were at their first entrance into all of the kindergartens located in the Longhua New District, and invited children's primary caregivers to fulfill a self-administered structured questionnaire. After excluding those who refused to respond or with incomplete required information, a total of 9,289 child-caregiver pairs were included for analysis. This study was approved by the Ethics Committee of the School of Public Health at Sun Yat-sen University (ethics clearance No.: 2015–016), and all participants provided their written informed consent.

### Data collection

Data were gathered through primary caregiver-reported structured questionnaires, which contained information regarding the parents' socio-demographic characteristics including education level, age at pregnancy, marital status, and family income, as well as the child's general information including gender, date of birth, single child or not, preterm birth (gestation < 36 weeks), low birth weight (the infant with birth weight ≤ 2,500 gram), family childcare arrangement, and conduct problem behaviors.

### Measurement of family childcare types

Family childcare types was determined from the question: “Who were the primary caregivers for the child at home during the following different ages: from child birth to 3 months, from 3 to 6 months, from 6 months to 1 year, from 1 to 2 years, and from 2 years till now, respectively?” The response choices were: “parents together,” “mother alone,” “mother with others (e.g., grandparents, relatives, friends, neighbors, or babysitters/nannies),” and “grandparents.” The primary childcare arrangement that we used was based on the type of childcare attended for the longest period of time.

Given that there were changes in the family childcare arrangement from child birth till the investigation date for some participants, in the present study, we further categorized all participants into the following five categories of family childcare types, including: “parents together,” “mother alone,” “mother with others,” “grandparents,” and “changing caregivers,” among which the first four categories consistently employed that particular kind of childcare arrangement throughout the whole investigated period.

### Measurement of caregiver-child interaction

Caregiver-child interaction was assessed based on the following questions: (1) “How often did the primary child caregivers perform specific activities with the target child from child birth to 1 year old, including: singing, chatting, playing games, body touch (e.g., hugging, kissing, caressing, etc.), and outdoor activities, respectively”; and (2) “How often did the primary child caregivers perform specific activities with the target child during 1–3 years old of the child, including: reading, singing, chatting, playing games, body touch (e.g., hugging, kissing, caressing, etc.), taking to parties, outdoor activities, and traveling, respectively”. The response choices were: “never,” “< 1 time/week,” “1–2 times/week,” and “more than 2 times/week,” which were accordingly allocated to a score of “0,” “1,” “2,” and “3,” respectively. We added up the score for each specific activity and then divided the total score by 13, to derive the final average score for measuring the overall level of caregiver-child interaction. The score for caregiver-child interaction ranged from 0 to 3, with a higher score indicating a higher level of caregiver-child interaction.

### Measurement of conduct problem behaviors

We assessed child conduct problem behaviors by a conduct problem (CP) subscale of the Conners' Parent Rating Scale-Revised (CPRS-48), an internationally disseminated and validated screening tool to assess behavioral difficulties in children aged between 3 and 16 years old (35). This tool had been translated into Chinese and showed a good reliability and validity as well (36). The Conners' CP measure comprises 13 items covering a range of defiant or aggressive behaviors. Each item was rated on a four-point scale according to the frequency of child's behaviors: never (score = 0), sometimes (score = 1), often (score = 2), and frequently (score = 3). The average score was then calculated, thereby we obtained a continuous variable ranging between 0 to 3, where a higher score indicated a higher level of conduct problem behaviors. In accordance with previous literature (36), we also transformed the average score into a categorical variable: children scoring larger than $\overset{-}{X}+2SD$ were classified as with conduct problem behaviors and were coded “1,” otherwise they were coded “0.” In the current study, the measurement of conduct problem behaviors was treated in both categorical and continuous formats for analysis.

### Covariates

The potential covariates included child gender and age, single child, preterm birth, low birth weight, parents' education level, age at the time of pregnancy, marital status and family income. The variables that were significant at *p* < 0.1 in univariate analyses or widely reported in the literature (e.g., child gender and age) were adjusted in the later multiple regression models.

### Statistical analysis

Means and standard deviations (SD) were used to describe continuous variables, and absolute frequencies and proportions were used for categorical variables. Chi-square tests were applied to compare the socio-demographic characteristics between children with and without conduct problem behaviors. The associations between family childcare arrangement and child conduct problem behaviors were examined by logistic regression models (when the outcome measurement took a categorical format) or linear regression models (when the outcome measurement took a continuous format), under the circumstances of both with and without adjustment for the aforementioned confounders. We also performed multiple linear or logistic regression models, whichever appropriate, to explore the associations of caregiver-parent interaction with family childcare types and child conduct problem behaviors.

**Figure 1:**
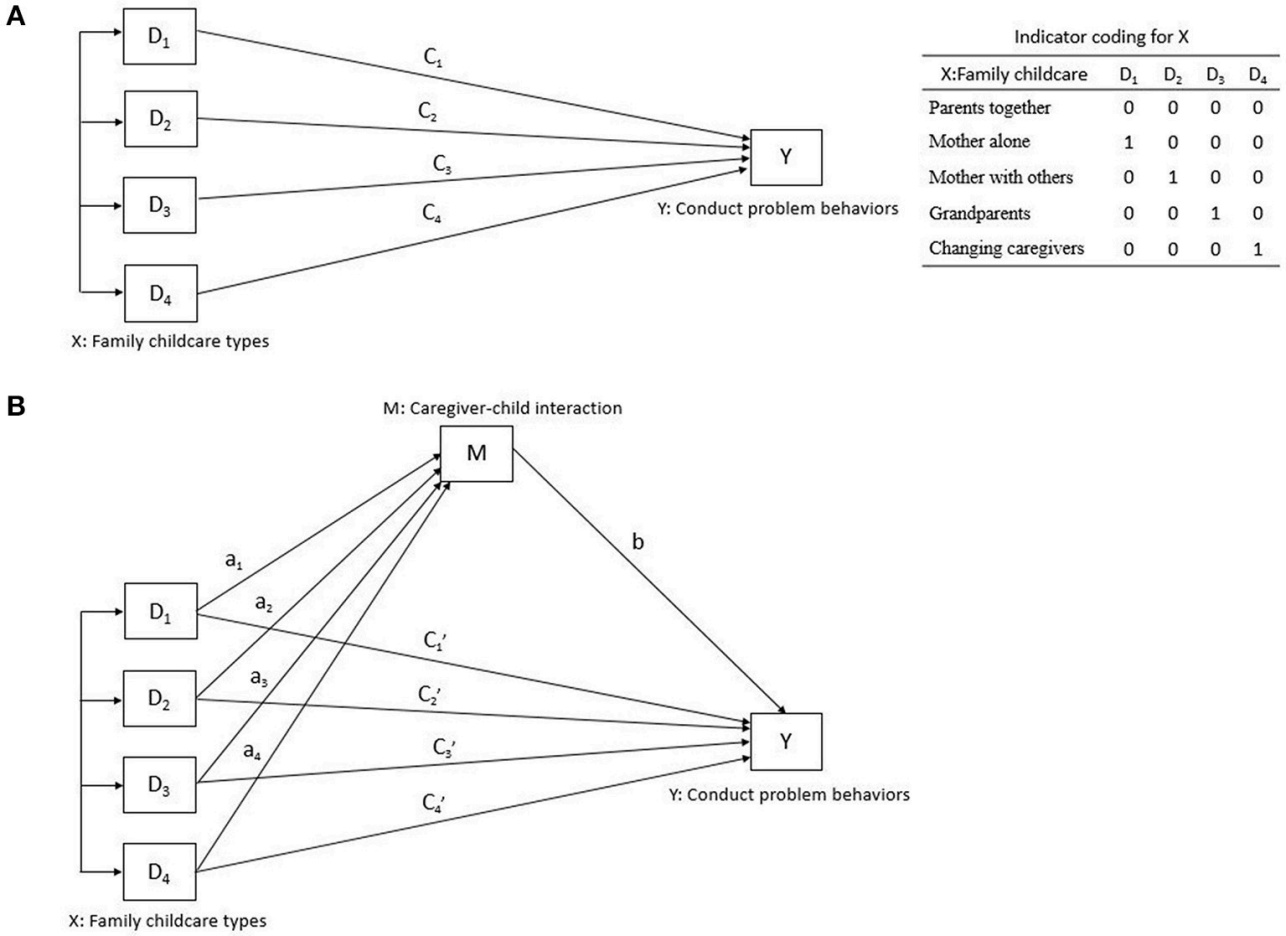
A simple mediation model in path diagram form. The upper figure **(A)**: Direct effect of X on Y; The lower figure **(B)**: Indirect effect of X on Y.

To further determine whether and to what extent caregiver-child interaction (Mediator, M) mediated the association between family childcare types (Independent variable, X) and child conduct problem behaviors (Dependent variable, Y), the simple mediation model (Figure 1) was conducted through a bootstrapping procedure with 5,000 resamples using Mplus (37). In this approach, we assessed the bias-corrected bootstrap confidence intervals (CI) of indirect effects, which were considered significant if the upper and lower bound of the 95% CI did not straddle zero. Given that the X (family childcare) in the study was a multicategorical variable, we obtained a relative indirect effect for each category (group D_i_) vs. the reference group, and evidence that at least one relative indirect effect was different from zero supported the conclusion that M mediated the effect of X on Y (38). All regression models adjusted for the aforementioned potential confounders. The proportion of mediation was then calculated as follows (39):

- When Y was taken as a categorical variable: $\frac{OR^{DE}\times \left(OR^{IE}-1\right)}{OR^{DE}\times \;OR^{IE}-1}$
- When Y was taken as a continuous variable: a_i_b/c_i_

where in Equation (i): OR^DE^ was the odds ratio of the coefficient of Xi on Y while holding M constant, and OR^IE^ was the exponent of the product by the partial coefficient of M on Y and X_i_ on M; and in equation (ii) a_i_ was the coefficient of X_i_ on Y, b was the coefficient of M on Y while controlling for X, c_i_ was the coefficient of X_i_ on Y, and a_i_b represented the relative indirect effects of X_i_ (being in group D_i_ relative to the reference group) on Y through M.

All analyses were conducted with SPSS version 22.0 (SPSS Inc., Chicago, IL), and a two-tailed *p* below 0.05 was considered statistically significant.

## Results

### Socio-demographic characteristics of the study population

The socio-demographic characteristics of participants are shown in Table 1. Of the total sample, 309 (3.3%) young children were identified as having conduct problem behaviors according to the recommended cut-off value. The included children were all aged 2.5–4 years, with about half boys (54.5%) and half girls (45.5%). Between the children with and without conduct problem behaviors, significant differences were observed in terms of parents' education level, parents' age at the time of maternal pregnancy, marital status, and family income. However, other characteristics were quite comparable between them including child age and gender, single child, preterm birth, and low birth weight.

**Table 1:** Socio-demographic comparisons of children with and without conduct problem behaviors.

**Variables**	**Total, *n* (%)**	**Conduct problem behaviors**, ***n*** **(%)**		
		**Yes**	**No**	***P* value^†^**
Total	9,289 (100.0)	309 (3.3)	8,980 (96.7)	-
Gender				0.816
Boy	5,062 (54.5)	166 (53.7)	4,896 (54.5)	
Girl	4,227 (45.5)	143 (46.3)	4,084 (45.5)	
Child age (month)				0.907
30–36	1,517 (16.3)	52 (16.8)	1,465 (16.3)	
37–42	4,935 (53.1)	166 (53.7)	4,769 (53.1)	
43–48	2,837 (30.5)	91 (29.4)	2,746 (30.6)	
Single child				0.231
Yes	3,427 (36.9)	124 (40.1)	3,303 (36.8)	
No	5,862 (63.1)	185 (59.9)	5,677 (63.2)	
Preterm birth				0.216
Yes	667 (7.2)	28 (9.1)	639 (7.1)	
No	8,622 (92.8)	281 (90.9)	8,341 (92.9)	
Low birth weight				0.415
Yes	447 (4.8)	18 (5.8)	429 (4.8)	
No	8,842 (95.2)	291 (94.2)	8,551 (95.2)	
Maternal education level				0.001^**^
≤ 12 years	4,584 (49.3)	182 (58.9)	4,402 (49.0)	
>12 years	4,705 (50.7)	127 (41.1)	4,578 (51.0)	
Paternal education level				0.002^**^
≤ 12 years	3,693 (39.8)	150 (48.5)	3,543 (39.5)	
>12 years	5,596 (60.2)	159 (51.5)	5,437 (60.5)	
Maternal age at pregnancy (year)				0.001^**^
< 23	1,607 (17.3)	73 (23.6)	1,534 (17.1)	
23–30	6,210 (66.9)	204 (66.0)	6,006 (66.9)	
>30	1,472 (15.8)	32 (10.4)	1,440 (16.0)	
Paternal age at pregnancy (year)				0.001^**^
< 23	1,477 (15.9)	72 (23.3)	1,405 (15.6)	
23–30	5,831 (62.8)	182 (58.9)	5,649 (62.9)	
>30	1,981 (21.3)	55 (17.8)	1,926 (21.4)	
Marital status				0.003^**^
Married	8,972 (96.6)	288 (93.2)	8,684 (96.7)	
Others	317 (3.4)	21 (6.8)	296 (3.3)	
Family income (Chinese RMB/month)				0.014^*^
0–9,999	4,476 (48.2)	173 (56.0)	4,303 (47.9)	
10,000–20,000	3,043 (32.8)	91 (29.4)	2,952 (32.9)	
>20,000	1,770 (19.1)	45 (14.6)	1,725 (19.2)	

† χ^2^ tests were used for categorical variables.

* *P < 0.05*,

** *P < 0.01*,

***P < 0.001.

### Association between family childcare types and child conduct problem behaviors

Table 2 presents results on the association between family childcare types and conduct problem behaviors in the young children. In the univariate analyses, results from both logistic regression (considering the outcome variable in categorical format) and linear regression (considering the outcome variable in continuous format) models revealed that, compared with children cared by parents together, those cared by mother alone, mother with others, or changing caregivers encountered significantly elevated risks for conduct problem behaviors, while those cared by grandparents showed non-significantly increased risks. In further multiple regression models with adjustment for potential confounders including child age, gender, parental education level, parental age at pregnancy, marital status, and family income, when compared with family childcare by parents together, children primarily cared by mother alone, mother with others, grandparents, or changing caregivers since birth were all significantly associated with higher risks of conduct problem behaviors at an early age of around 3 years (adjusted ORs ranged from 2.18 to 2.55, and adjusted βs ranged from 0.043 to 0.073; all *p* < 0.05).

**Table 2:** Logistic and linear regression models on the association between family childcare types and conduct problem behaviors in young children.

**Family childcare types**	**Total, *n* (%)**	**Conduct problem behaviors**					
		**Logistic regression (Taken as a categorical variable)**			**Linear regression (Taken as a continuous variable)**		
		**Case**, ***n*** **(%)**	**Crude model**^†^	**Adjusted model**	**Score, Mean** ± **SE**	**Crude model**^†^	**Adjusted model**
			**Crude OR (95% CI)**	**Adjusted OR (95% CI)**		**Crude** β **(95% CI)**	**Adjusted** β **(95% CI)**
Parents together	1228 (13.2)	21 (1.7)	Ref	Ref	0.470 ± 0.009	Ref	Ref
Mother alone	347 (3.7)	14 (4.0)	2.42 (1.22, 4.80)^*^	2.53 (1.27–5.04)^**^	0.523 ± 0.019	0.053 (0.013, 0.093)^**^	0.059 (0.019, 0.099)^**^
Mother with others (except father)	1530 (16.5)	56 (3.7)	2.18 (1.32, 3.63)^**^	2.55 (1.52–4.27)^***^	0.523 ± 0.009	0.053 (0.028, 0.078)^***^	0.063 (0.037, 0.088)^***^
Grandparents	320 (3.4)	10 (3.1)	1.85 (0.86, 3.98)	2.18 (1.01–4.71)^*^	0.502 ± 0.019	0.032 (−0.009, 0.074)	0.043 (0.002, 0.085)^*^
Changing caregivers	5864 (63.1)	208 (3.5)	2.11 (1.34, 3.33)^**^	2.33 (1.47–3.69)^***^	0.539 ± 0.004	0.069 (0.048, 0.089)^***^	0.073 (0.051, 0.094)^***^

† Model was unadjusted for any confounding variables.

‡*Model was adjusted for confounders including child age, gender, parental education level, parental age at pregnancy, marital status, and family income*.

* *P < 0.05*,

** *P < 0.01*,

*** *P < 0.001*.

### Association of caregiver-child interaction with family childcare types and child conduct problem behaviors

Table 3 depicts the association of caregiver-child interaction with family childcare types and child conduct problem behaviors. Compared with the childcare arrangement by parents together, childcare by grandparents was found to be significantly associated with a largest degree of decrease in the caregiver-child interaction level after adjusting for potential confounders (β = −0.168, 95%CI: −0.210, −0.126). Childcare by mother alone (β = −0.070, 95%CI: −0.110, −0.029) or changing caregivers (β = −0.067, 95%CI: −0.089, −0.046) were also associated with significant decreases in the levels of caregiver-child interaction, while the childcare by mother with others only non-significantly decreased the caregiver-child interaction (β = −0.024, 95%CI: −0.050, 0.002). Besides, an increasing level of caregiver-child interaction was found to be significantly associated with decreased risks of conduct problem behaviors in children, after controlling for potential confounders (OR = 0.45, 95%CI: 0.34, 0.59; β = −0.118, 95%CI: −0.138, −0.099).

**Table 3:** Association of caregiver-child interaction with family childcare types and child conduct problem behaviors.

**Characteristics**	**Caregiver-child interaction**		**Conduct problem behaviors**	
	**Mean ± SD**	**β (95% CI)^†^**	**Taken as a categorical variable: OR (95% CI)^†^**	**Taken as a continuous variable: β (95% CI)^†^**
Family childcare types				
Parents together	2.59 ± 0.32	Ref		
Mother alone	2.49 ± 0.39	−0.070 (−0.110, −0.029)^***^		
Mother with others	2.55 ± 0.36	−0.024 (−0.050, 0.002)		
Grandparents	2.46 ± 0.44	−0.168 (−0.210, −0.126)^***^		
Changing caregivers	2.53 ± 0.35	−0.067 (−0.089, −0.046)^***^		
Caregiver-child interaction			0.45 (0.34, 0.59)^***^	−0.118 (−0.138, −0.099)^***^

† Model was adjusted for confounders including child age, gender, parental education level, parental age at pregnancy, marital status, and family income.

*** *P < 0.001*.

### Mediation effect of caregiver-child interaction on the association between family childcare types and child conduct problem behaviors

The mediation effect of caregiver-child interaction on the association between family childcare types and child conduct problem behaviors is illustrated in Table 4. Results showed that after controlling for aforementioned confounders, all of the following family childcare types (by mother alone, or by grandparents, or by changing caregivers) relative to the reference group of childcare by parents together showed significant relative indirect effects on child conduct problem behaviors through caregiver-child interaction (95% bias-corrected bootstrap CIs for all of their corresponding relative indirect effects did not overlap zero, regardless of whether taking the dependent variable as a categorical format or continuous format), and the relative proportion of mediation for these conditions were 8.9% (or 13.7%), 23.4% (or 44.9%), and 9.0% (or 10.8%), respectively. However, the mediation of caregiver-child interaction on the effect of being in the group of childcare by mother with others relative to the reference group of by parents together on child conduct problem behaviors was non-significant (when Y was taken as a categorical variable: 0.019, 95%CI = −0.005, 0.039; when Y was taken as a continuous variable: 0.003, 95%CI = −0.0002, 0.006).

**Table 4:** Mediation effect of caregiver-child interaction on the association between family childcare types and child conduct problem behaviors.

**Characteristics**	**Conduct problem behaviors (Dependent variable, Y)**		**Mediation Effect**	
	**Model 2^†^: X → Y**	**Model 3^†^: X+M → Y**	**Relative indirect effect (95% CI)**	**Proportion of mediation, %**
	**Coefficient (95% CI)**	**Coefficient (95% CI)**		
**Y TAKEN AS A CATEGORICAL VARIABLE**				
**Family childcare types (Independent Variable, X)**				
Parents together (Ref)	Ref	Ref		
Mother alone (D_1_)	2.53 (1.27–5.04)^**^	2.36 (1.18, 4.72)^*^	**0.055 (0.014, 0.093)**	8.9%
Mother with others (D_2_)	2.55 (1.52–4.27)^***^	2.52 (1.50, 4.22)^***^	0.019 (−0.005, 0.039)	NA
Grandparents (D_3_)	2.18 (1.01–4.71)^*^	1.86 (0.85, 4.04)	**0.133 (0.064, 0.192)**	23.4%
Changing caregivers (D_4_)	2.33 (1.47–3.69)^***^	2.22 (1.40, 3.51)^**^	**0.053 (0.025, 0.077)**	9.0%
Caregiver-child interaction (Mediator, M)		0.45 (0.34, 0.60)^***^		
**Y TAKEN AS A CATEGORICAL VARIABLE:**				
**Family childcare types (Independent Variable, X)**				
Parents together (Ref)	Ref	Ref		
Mother alone (D_1_)	0.059 (0.019, 0.099)^**^	0.051 (0.011, 0.091)^*^	**0.008 (0.003, 0.014)**	13.7%
Mother with others (D_2_)	0.063 (0.037, 0.088)^***^	0.060 (0.034, 0.085)^***^	0.003 (−0.0002, 0.006)	NA
Grandparents (D_3_)	0.043 (0.002, 0.085)^*^	0.024 (−0.018, 0.065)	**0.019 (0.013, 0.027)**	44.9%
Changing caregivers (D_4_)	0.073 (0.051, 0.094)^***^	0.065 (0.044, 0.086)^***^	**0.008 (0.005, 0.011)**	10.8%
Caregiver-child interaction (Mediator, M)		−0.116 (−0.136, −0.096)^***^		

† *Model was adjusted for confounders including child age, gender, parental education level, parental age at pregnancy, marital status, and family income*.

*‡Relative indirect effect represented the indirect effect on Y via M of being in group Di relative to the reference group*.

* *P < 0.05*,

** *P < 0.01*,

*** *P < 0.001; Bold fonts denote statistical significance at 0.05 level. NA: The indirect effect was not statistically significant*.

## Discussion

To the best of our knowledge, this is the first study examining the association between family childcare and conduct problem behaviors in Chinese preschool children with caregiver-child interaction as a potential mediator. Findings from the current study support that compared with stable childcare by parents together since child birth, other family childcare types (including mother alone, mother with others expect father, grandparents, and changing caregivers) are all associated with augmented risks for conduct problem behaviors in preschool children. In addition, caregiver-child interaction plays a mediating role in the above associations, especially mediating a sizeable proportion of the effect of being cared by grandparents vs. by both parents on child conduct problem behaviors. Its mediation effect was only non-significant on the effect of being cared by mother with others relative to care by parents together.

An emerging body of research has examined the associations between family childcare and child behavioral development, but their conclusions still remained controversial. Several studies demonstrated a protective effect of parental care on cognitive outcomes or externalizing behavior problems in children (15–17); while another study found that compared with parental care, children receiving non-relative and center care showed no increase in relative risk of severe externalizing behaviors (14). Most studies found that non-maternal childcare would impose adverse impact on child psychobehaviroal development, such as increasing aggression, troublesome and oppositionality, etc. (40–42); however, the above association might not be true for those children who were from poor family with low-educated mothers but were receiving non-maternal care (25). Father involvement in childcare was widely supported by prior research as associated with improvement for psychological, cognitive, and academic development of children (14, 18, 19). Childcare from grandparents (20, 21) or relatives (14) were found to be significantly associated with more behavioral problems in children. Besides, the majority of studies consistently pointed out that children who experienced multiple and concurrent childcare arrangements displayed more internalizing and externalizing behavior problems and fewer prosocial behaviors (12, 22–24); however, there were also a few studies supporting that childcare changes leading to higher quality or more developmentally appropriate care, such as transitioning from low socio-economic family childcare or low-educated mother's care to high quality child care (43) and transitioning from in-home care to center-based care during the preschool years (44, 45), were beneficial to improve outcomes including better school readiness, improved cognitive performance, and decreased behavioral problems. In the present study, our findings are consistent with several previous studies, which underscored the importance of stable childcare engagement from both parents during early childhood. Our study shows that family childcare types other than by parents together (i.e., mother alone, mother with others, grandparents, and changing caregivers) are all associated with elevated risks for conduct problem behaviors in young children. Discrepancies among previous studies may be owing to extensive differences in the social and cultural patterns or the demographic characteristics, as well as the varied definitions of childcare across studies.

The following explanations may account for the above association found in our study. That is, childcare by parents together represent a quite complementary and balanced caring style, with mothers providing more nurturing care and emotional support while fathers providing more playful and physically stimulating interactions (18, 30, 31). Such care itself may also imply a harmonized and well-functioned family environment or parental psychopathology (46), which may ultimately improve child development. The most current research based on animal studies of mice even demonstrated that early life experience with maternal care could alter DNA methylation at YY1 binding sites implicated in L1 activation, and would then affect expression of the *de novo* methyltransferase DNMT3a which appear to have substantial functional effects in the brain development and behavior (47, 48). Meanwhile, considerable evidence from Feldman et al. demonstrated that early parent-infant interactions are critical for shaping the children's development of moral stance, neurobehavioral, cognitive and social-emotional outcomes in the later years or across the lifespan (49–51). However, when it comes to family childcare types other than parents together, for example, if mothers take the responsibility of childcare all by themselves alone, they may be overwhelmingly busy and unable to provide quality care. Childcare by mother with others could ensure enough functioning of mothers, yet lacks the involvement of paternal functioning and perhaps includes more conflict or competition in terms of parenting, education and life style between mother and other caregivers than mother and father (30). Grandparental care is often committed to mainly caring for the child's physiological demands, while ignoring necessary parenting, interaction and emotional caring (21, 29). Changes in care arrangement disrupt children's already attached or emerging relationships with caregivers, which in turn, may destroy the secure attachment and positive interactions with particular caregiver and may impede children's social-emotional adjustment and development, especially during infancy or young childhood (12, 52, 53). However, despite of the above presumed reasons, the exact explanation for the differences in associations between different childcare types and child psycho-behavioral development remains uncertain and requires further in-depth research.

Based on prior studies, different family childcare types are commonly known to be associated with varieties in caregiver-child interaction. For example, parents were more involved in interacting activities that actively stimulated the children's intellect and playfulness, while grandparents, who often devoted themselves in providing the basic caring in terms of dieting, dressing, sleeping and other living conditions, were less playfully interactive with the children that they cared (21, 29). In addition, mothers were often very emotionally supportive but less physically active, whereas fathers tended to spend more time in playing with their children or participating in adventurous and amusing interactive activities (18, 30, 31). Our findings also confirm that childcare by parents together is likely to have a highest level of caregiver-child interaction, followed by mother with others, changing caregivers, mother alone, and grandparents. In addition, previous research also demonstrated that more involvement in caregiver-child interaction such as reading and outdoor play would be likely to result in better cognitive and behavioral development, language acquisition and social abilities in young children (28, 32–34). In accordance with the previous findings, our study observe that an elevated level of caregiver-child interaction is associated with lower risks for child conduct problem behaviors. The positive association between caregiver-child interaction and child behavioral outcomes may be owing to the fact that: a more active engagement in interaction with caregiver may help the child acquire ever-increasing training in communication, social adaption, intellectual stimulation and so on, all of which are likely to link to the healthy and all-round psychological development of child.

Extensive research has indicated that child conduct problem behaviors may be attributable to a complex combination of genetic and environmental factors involving child, family and community (3, 4, 6, 10, 11); however, the underlying mechanisms remain poorly investigated and understood. In the present study, we observe that caregiver-child interaction medicates 23.4% (or 44.9%, the difference in percentage was owing to the categorical or continuous format of the outcome variable that we used) of the effect of being cared by grandparents vs. by parents together on increasing the conduct problem risks. The proportion of mediation effect turns smaller in size but remains significant, when comparing childcare by mother alone (or by changing caregivers) with parents together. Our results indicate that caregiver-child interaction serve as a potential mediator in the association of family childcare with child conduct problem behaviors, especially mediating a sizeable proportion of the effect of being cared by grandparents vs. by both parents. To our best knowledge, there was no prior study that had ever explored the mediating role of caregiver-child interaction in the relationship of family childcare with child behavioral development. Our findings highlight the importance of maintaining stable parental engagement in childcare or increasing active caregiver-child interactions, thereby possibly improving child behavioral and psychological development.

Several limitations should be acknowledged when interpreting the findings in the study. First, we employed a cross-sectional study design that could not determine the causal relationship between childcare and child conduct problems. Results from the present study should be interpreted cautiously, and it is warranted that we conduct larger research in the future to investigate the exact causality and mediation effect. Second, the data was retrospectively collected using a structured questionnaire completed by the primary caregivers, which might result in an information bias. Thus interpretation of the results would require cautions and caveats. Third, clinical diagnosis of conduct problems in children was unavailable in this study, considering the infeasibility to diagnose by interview or direct observation in a large-scale survey. Instead, our study used the Conners' Conduct Problem Subscale, which was widely accepted in epidemiological research and proved to show good reliability and validity in the Chinese population. Fourth, given that formal childcare is rarely used in China, we could not compare the role of formal vs. informal childcare, which may vary in their effects on the development and progression of child's conduct problems. Fifth, we did not collect detailed information regarding the quality of childcare arrangement and caregiver-child interaction, which prevented us from understanding the more precise and sophisticated association between childcare and child development. Last, our study did not involve children who were absent in kindergarten. However, conduct problem behaviors may be more serious among that population (although that population size could be very minor).

## Conclusions

Findings from the current study support that family childcare types other than by parents together are associated with elevated risks for conduct problem behaviors in young children. Caregiver-child interaction functions as a potential mediator for the above associations, especially mediating a sizeable proportion of the effect of being cared by grandparents vs. by both parents on child conduct problem behaviors. Our study provides encouraging insights that parents being involved together in family childcare are likely to introduce beneficial effects on the child's psycho-behavioral development, and also adds to the scarce knowledge concerning caregiver-child interaction as a plausible mechanism underlying the associations between family childcare types and child conduct problem behaviors.

## Data availability

The datasets for this manuscript are not publicly available because: part of the data is included in other manuscripts under preparation. Requests to access the datasets should be directed to Wei-Qing Chen: chenwq@mail.sysu.edu.cn.

## Author contributions

W-QC, C-AW, LL, X-NY, G-MW, DS, D-XX, JJ, and YJ initiated and designed the study. W-QC led the research training. C-AW led the field investigation. LL, X-NH, G-MW, DS, D-XX, and HJ took part in the investigation team. LL, LF, and W-QC analyzed and interpreted the data. LL, LF, and W-QC wrote the manuscript. X-YH and W-QC revised it with suggestions from all authors.

### Conflict of interest statement

The authors declare that the research was conducted in the absence of any commercial or financial relationships that could be construed as a potential conflict of interest.
